# Self-Refinement of Auxiliary-Field Quantum Monte Carlo via Non-Orthogonal Configuration Interaction

**DOI:** 10.1021/acs.jctc.5c00127

**Published:** 2025-04-28

**Authors:** Zoran Sukurma, Martin Schlipf, Georg Kresse

**Affiliations:** † University of Vienna, Faculty of Physics, Kolingasse 14-16, A-1090 Vienna, Austria; ‡ VASP Software GmbH, Berggasse 21/14, 1090 Vienna, Austria

## Abstract

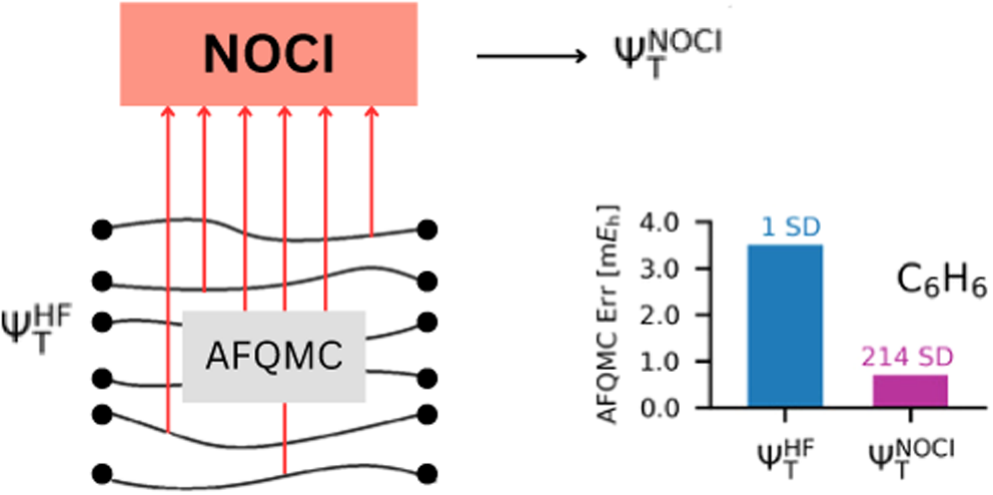

For optimal accuracy, auxiliary-field quantum Monte Carlo (AFQMC) requires trial states consisting of multiple Slater determinants. We develop an efficient algorithm to select the determinants from an AFQMC random walk eliminating the need for other methods. When determinants contribute significantly to the nonorthogonal configuration interaction energy, we include them in the trial state. These refined trial wave functions significantly reduce the phaseless bias and sampling variance of the local energy estimator. With 100 to 200 determinants, we lower the error of AFQMC by up to a factor of 10 for second-row elements that are not accurately described with a Hartree–Fock trial wave function. For the HEAT set, we improve the average error to within chemical accuracy. For benzene, the largest studied system, we reduce AFQMC error by 80% with 214 Slater determinants and find a 10-fold increase of the time to solution. We show that phaseless errors prevail in systems with static correlation or strong spin contamination. For such systems, improved trial states enable stable free-projection AFQMC calculations, achieving chemical accuracy even in the strongly correlated regime.

## Introduction

Accurate large-scale applications of the post-Hartree–Fock methods have become feasible with advances in computing power.^1−3^ Coupled-cluster singles, doubles, and perturbative triples (CCSD(T))^4,5^ and fixed-node diffusion Monte Carlo (FN-DMC)^6−8^ are two of the most prominent methods, offering an excellent balance between accuracy and computational cost. However, recent studies^1,2^ indicate increasing discrepancies between the two methods for large noncovalent complexes. For example, the discrepancy for a C_60_ buckyball inside a [6]-cycloparaphenyleneacetylene ring (132 atoms) amounts to 7.6 kcal/mol after ruling out all stochastic uncertainties. Such large discrepancies lead to mistrust in both methods, especially as each is considered highly reliable for predicting noncovalent interaction energies. Schäfer et al.^3^ pointed out that CCSD(T) may not be sufficiently accurate and that correcting the perturbative triples^9^ improves interaction energies at the cost of less accurate total energies.

Auxiliary-field quantum Monte Carlo (AFQMC)^10−12^ has emerged as a promising alternative to CCSD(T), gaining increasing popularity in recent years.^13−26^ Several key factors contribute to the growing popularity of AFQMC: (i) its formulation in second quantization, enabling straightforward application on top of the mean-field methods, (ii) its favorable computational scaling— for AFQMC compared to  for CCSD(T), and (iii) its demonstrated accuracy. In its simplest formulation with a single-determinant trial wave function, AFQMC accuracy typically falls between that of CCSD and CCSD(T).^18,22,23,25^ The main source of error in AFQMC is the phaseless approximation^10,11,23,27,28^ introduced to control the Fermionic phase problem. Additionally, AFQMC requires many steps to reduce the sampling variance leading to a large prefactor of the quartic scaling. Together, these factors continue to hinder efficient large-scale AFQMC calculations.

More accurate trial wave functions reduce the phaseless error and sampling variance and are thus a promising route toward more accurate AFQMC. Particle-hole multi-Slater determinants (PHMSD) are the most convenient choice for constructing such trial wave functions. Since the computational cost of a naive implementation scales linearly with the number of determinants, many groups have developed fast local energy evaluation methods for PHMSD trial wave functions.^26,29−33^ Shee et al.^29^ were the first to report an efficient evaluation of local energies over PHMSD trial wave functions using the Sherman–Morrison–Woodbury formula.^34^ Later, Shi and Zhang^31^ optimized the algorithm further, performing calculations with 10,000 determinants and achieving a 60-fold speedup compared to the naive implementation. Their algorithm scales as , where *N*_g_ is the number of auxiliary fields, *N* is the number of orbitals, *N*_e_ is the number of electrons, and *N*_d_ is the number of determinants. The notation *Ñ*_d_ indicates sublinear scaling with the number of determinants. Mahajan et al.^30,32^ applied the generalized Wick’s theorem^35^ to design an algorithm that scales as . They reported calculations using 10,000 determinants with only a 3-fold increase in computational cost compared to the single-determinant case.^33^ Configuration-interaction-singles-doubles (CISD) trial wave functions containing up to half a billion of PHMSDs represent the largest determinantal expansion used with AFQMC to date.^26^

In addition to the PHMSD wave functions, nonorthogonal multi-Slater determinants (NOMSD) provide an appealing alternative, often requiring fewer determinants than the orthogonal case. Borda et al.^36^ performed AFQMC calculations using up to 250 NOMSD determinants. They generated the trial wave functions using the few-determinant approach^37−39^ and the resonating Hartree–Fock method.^40,41^ For these wave functions, the local energy evaluation scales linearly with the number of determinants, which limits its practical application as the number of determinants grows. Alternative choices for the trial state of AFQMC simulations are general Hartree–Fock (GHF) wave functions,^42^ matrix-product states,^43^ and states obtained using quantum computers.^44−47^

In this work, we use the AFQMC method itself to generate candidate determinants for the nonorthogonal Configuration interaction (NOCI) and refine the AFQMC trial wave functions. We carefully design the selection process to achieve the most compact NOCI expansion. During each epoch of determinant selection, we perform a short AFQMC random walk and select determinants that (1) have sufficiently negative local energies, (2) exhibit a small overlap with the currently selected NOCI wave function, and (3) contribute significantly to the correlation energy. We demonstrate that the resulting method, denoted as AFQMC/NOCI, substantially reduces phaseless errors and the AFQMC sampling variance. Reducing sampling variance often offsets the additional computational cost introduced by the NOCI trial wave function. We note that the idea of self-refinement in AFQMC is not new. Qin et al.^48^ tuned independent-electron calculations to reproduce the same density matrix as the constrained-phase AFQMC,^49,50^ thereby generating an optimized trial determinant for subsequent AFQMC calculations.

The remainder of the paper is structured as follows: we briefly recapitulate the AFQMC and NOCI methods and describe the selection process in detail in the next section. Then, the practical implementation details of the selection process that yields the most compact NOCI expansion are described in the section Implementation Details. We validate our approach through various applications on small molecular systems in the Results and Discussion section. Finally, we summarize our work and possibilities for future developments in the Conclusions section.

## Theoretical Background

We begin this section by describing the AFQMC random walk. Then, we describe the NOCI method and the selection process.

### AFQMC Random Walk

The AFQMC algorithm extracts the many-body ground state |Φ⟩ from the initial state |Φ_0_⟩ by applying long imaginary time propagation
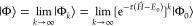
1where τ is the time step, *E*_0_ is the best estimate of the ground state energy, and *Ĥ* is the many-body Hamiltonian. At a given time step *k*, we approximate the ground state wave function |Φ_*k*_⟩ as the weighted average of *N*_*w*_ walkers
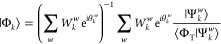
2where *w* enumerates *N*_*w*_ walkers and |Φ_T_⟩ is a trial wave function that guides walkers to the most important regions of the configuration space. Each walker is represented by a single Slater determinant |Ψ_*k*_^*w*^⟩, a real-valued weight *W*_*k*_^*w*^ and a phase θ_*k*_^*w*^, initialized as
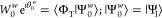
3|Ψ_I_⟩ is the initial determinant, usually chosen to be the Hartree–Fock determinant. With these ingredients, the AFQMC random walk is defined as
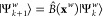
4
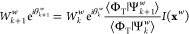
5The explicit form of the propagator *B̂*(**x**) depends on the specific realization of the Trotter decomposition^51^ and the Hubbard–Stratonovich transformation.^52,53^ We showed in our recent work^54^ that
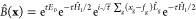
6yields minimal time-step errors. The vector **x** = {*x*_*g*_}_*g* = 1_^*N*_*g*_^ is a random vector drawn from the standard normal distribution, and the vector **f** = {*f*_*g*_}_*g* = 1_^*N*_*g*_^ represents the centers of the shifted Gaussian distribution. They are chosen as
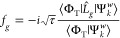
7to minimize the accumulated phase of the walker. The importance sampling reweighting factor *I*(**x**) is the ratio between standard normal and shifted Gaussian distributions
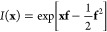
8The energy of the system is the weighted average of the local energies
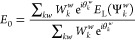
9where the local energy is computed using the nonorthogonal Wick theorem^55^
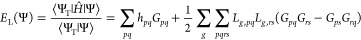
10The interstate reduced one-body density matrix *G* is given by
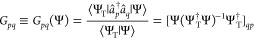
11The previous two equations apply to the single-determinantal |Ψ_T_⟩, and can be easily generalized for the multideterminantal wave function |Φ_T_⟩.^31^

### NOCI Selection Process

Following from eqs 1, 2, and 4, the exact ground-state wave function is approximated by an integral over random fields
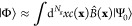
12where *c*(**x**) is an unknown amplitude function representing |Φ⟩, and *B̂*(**x**)|Ψ_0_⟩ denotes nonorthogonal Slater determinants for different **x** vectors. Here, |Ψ_0_⟩ is a reference determinant, usually a Hartree–Fock wave function. This approximation would become an equality if *B̂*(**x**^1^)*B̂*(**x**^2^) = *B̂*(**x**^1^ + **x**^2^). The discretized form of the previous equation serves as the basis for the NOCI expansion
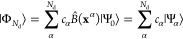
13The selection process extracts *N*_d_ Slater determinants |Ψ_α_⟩ from the AFQMC random walk and determines the optimal coefficients *c*_α_ solving the NOCI equation
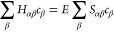
14where *E* is an upper bound to the true ground state energy *E*_0_. The Hamiltonian and the overlap matrix elements
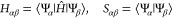
15are computed using the nonorthogonal Wick theorem,^55^ similar to eq 10.

We divide the selection process into three stages: (1) preselection based on AFQMC local energies, (2) a metric test, and (3) an energy test, both typically employed in NOCI.^56,57^

#### AFQMC Preselection

AFQMC walkers with low negative local energies tend to have large weights, small overlap with the trial wave function |Ψ_T_⟩, and significant contribution to the total AFQMC energy (eq 9). This makes them suitable candidates for inclusion in the NOCI expansion. For a given ensemble of walkers in |Φ_*k*_⟩ defined in eq 2, we calculate the mean local energy
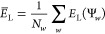
16and the standard deviation
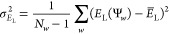
17Walkers with local energies satisfying
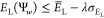
18are advanced to the further NOCI selection. λ is a numeric parameter that needs to be adjusted to obtain an optimized trial state. This step requires only local energy information, making it computationally simpler than the subsequent two tests.

#### Metric Test

We define the projection operator
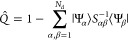
19that projects the candidate determinant |Ψ⟩ on the space orthogonal to the space spanned by *N*_d_ determinants in |Φ_*N*_d__⟩. We note that the projection operator *Q̂* is Hermitian *Q̂*^†^ = *Q̂* and idempotent *Q̂*^2^ = *Q̂*. The projection *Q̂*|Ψ⟩ is not a single Slater determinant and can be interpreted as a residual vector to the space spanned by determinants in |Φ_*N*_d__⟩. We define a metric threshold μ and require
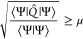
20This constraint effectively eliminates linear dependencies between Slater determinants in |Φ_*N*_d__⟩ and prevents the NOCI overlap matrix *S*_αβ_ from becoming singular.

#### Energy Test

The energy test measures the contribution of the candidate determinant |Ψ⟩ to the total energy of |Φ_*N*_d__⟩. We could compute the exact contribution with the NOCI eq 14, but solving it for every candidate determinant is computationally too expensive. Instead, we determine coefficients of a smaller variational problem with only two degrees of freedom
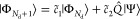
21by solving
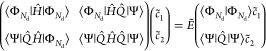
22The variational energy *Ẽ* is the approximation of the full variational energy, and it is sufficiently accurate for the selection process. A candidate determinant |Ψ⟩ passes the energy test if
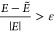
23for an energy threshold ε.

If a candidate determinant |Ψ⟩ meets all three criteria, it is added to the NOCI expansion, and the NOCI eq 14 is solved again to update the coefficients *c*_α_.

## Implementation Details

In this section, we study the impact of all parameters on the efficiency of the optimized NOCI selection algorithm. We benchmark the algorithm on the O_2_ molecule using the cc-pVDZ basis set and frozen-core approximation, a representative system for which AFQMC/HF performs poorly.^23^

Inspired by Chen et al.,^58^ we constrain the AFQMC random walk during the NOCI selection process to the first 100 Cholesky vectors for all systems considered in this work. This is achieved by setting *L̂*_*g*_ = 0 for *g* > 100 in eq 6. While not essential, this constraint accelerates the selection process and produces more compact NOCI wave functions.

We optimize the trial wave function over *N*_ξ_ epochs. From one epoch to the next, we systematically decrease the energy threshold ε
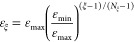
24for ξ = 1, 2, ···, *N*_ξ_. As a consequence, we add only determinants with a large impact on the energy in early epochs. In later epochs, where the trial wave function is already more accurate, we also add determinants with a smaller impact on the energy. In each epoch, we must provide a sufficiently large number of candidate determinants for the energy test to ensure convergence of the selection process with respect to the current energy threshold ε_ξ_. We use a fixed number of walkers *N*_*w*_ = 6,400 and *N*_*k*_ = 100 time steps of length τ = 0.05*E*_h_^–1^. This ensures convergence for all reasonable choices of λ and μ. After each time step, we update the NOCI wave function with determinants that meet all three criteria given in the section NOCI Selection Process. At the end of the epoch, we update the trial wave function for the next one.

**Figure 1:**
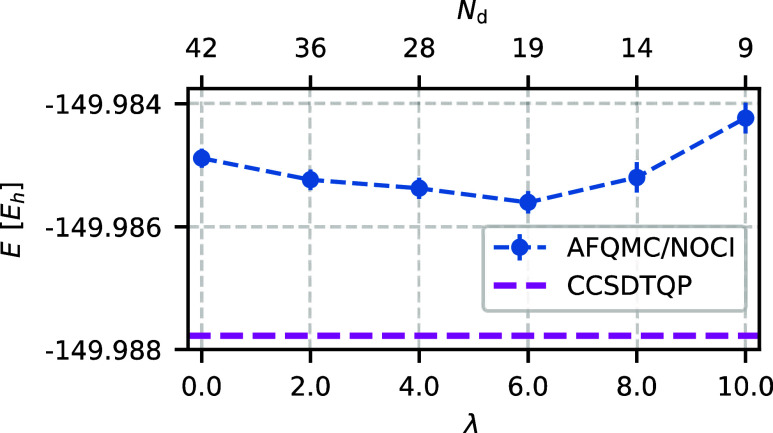
AFQMC/NOCI energy (blue dots) does not depend strongly on the preselection parameter λ, with the deviation from the reference CCSDTQP energy (magenta line) being nearly constant. Small λ values increase the number of determinants *N*_d_, whereas large values yield a larger sample variance. We select λ = 4.0 as a balanced choice between accuracy and efficiency. The system under consideration is O_2_ in the cc-pVDZ basis set. Other selection parameters are chosen as specified in Table 1, with ε_min_ = 10^–5^.

The parameters λ for the preselection and μ for the metric test are constant across all epochs because they have a smaller impact on the accuracy of the method. Let us consider λ first. Figure 1 shows the AFQMC energy with a NOCI trial wave function (AFQMC/NOCI energy) as a function of the parameter λ for otherwise fixed parameters. Small λ values increase the number of determinants without significant improvement of the energy. Conversely, large λ values are overly restrictive and may compromise selection accuracy. We find that any λ ∈ [2.0, 6.0] leads to a balanced and converged selection. We choose λ = 4.0 because it consistently selects up to one percent of the walkers as candidates.

**Figure 2:**
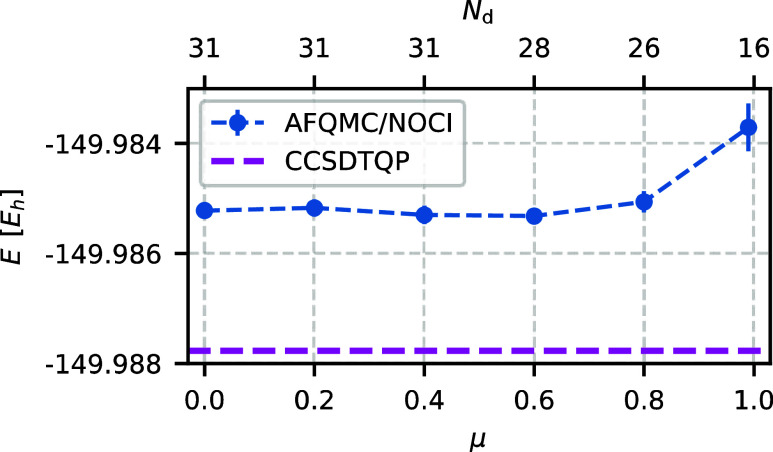
AFQMC/NOCI energy (blue dots) converges rapidly as the metric parameter μ decreases. The number of determinants *N*_d_ remains nearly constant as long as μ is not too large. We use μ = 0.6 throughout this work. The system under consideration is O_2_ in the cc-pVDZ basis set. Other selection parameters are chosen as specified in Table 1, with ε_min_ = 10^–5^.

Next, we consider the dependence of the AFQMC/NOCI energy on the parameter μ (Figure 2). For a wide range of values, μ has little impact on the number of determinants and the AFQMC/NOCI energy. Too large values of μ remove too many candidates, leading to a significant increase in the variance. In other systems, we also observed that small values of μ may lead to linear-dependency issues. We set μ = 0.6 as a balanced compromise.

Introducing epochs to dynamically adjust the energy test increases the number of adjustable parameters of the selection process. However, as we will show, this effort is justified because the lower bound ε_min_ for the energy is the single most important convergence parameter. The upper bound ε_max_ used in the first epoch and the number of epochs *N*_ξ_ do not significantly alter the results. ε_max_ should be large enough that only a few determinants are selected in the first epoch. For all systems considered in this work, we find that 10^–4^ ≤ ε_max_ ≤ 4 × 10^–4^ is an appropriate choice.

**Figure 3:**
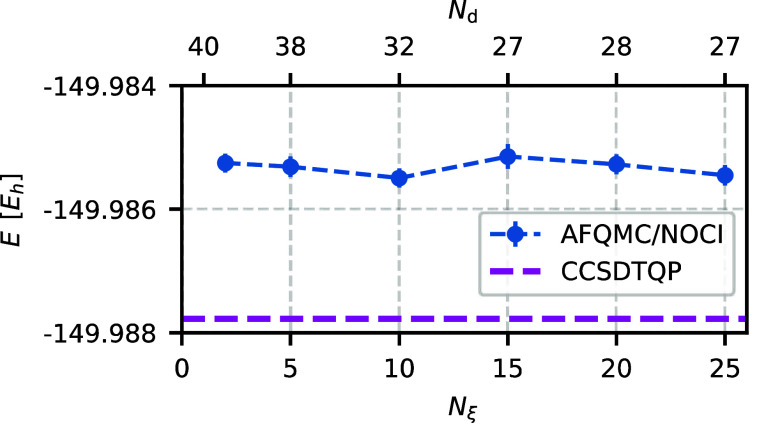
AFQMC/NOCI energy (blue dots) depends weakly on the number of epochs *N*_ξ_ for fixed ε_min_, ε_max_ values. However, the number of determinants *N*_d_ decreases as *N*_ξ_ increases. For typical ε_min_ and ε_max_ values, we use *N*_ξ_ = 10 throughout this work. The system under consideration is O_2_ in the cc-pVDZ basis set. Other selection parameters are chosen as specified in Table 1, with ε_min_ = 10^–5^.

The number of epochs *N*_ξ_ controls the final number of determinants in |Ψ_*N*_d__⟩ for fixed ε_min_ and ε_max_ values. Figure 3 shows the AFQMC/NOCI energy as a function of *N*_ξ_. For a small number of epochs, ε decreases rapidly toward ε_min_ and more determinants are selected. As the number of epochs increases, the number of determinants decreases without compromising accuracy. Beyond a certain *N*_ξ_ value, the number of determinants no longer changes, while the computational cost increases linearly with *N*_ξ_. As a reasonable compromise, we fix *N*_ξ_ = 10 throughout this work.

**Figure 4:**
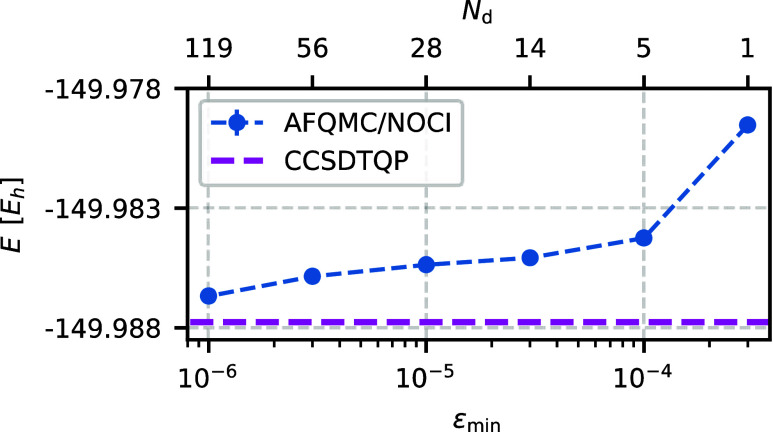
AFQMC/NOCI energy (blue dots) converges toward the reference CCSDTQP energy (magenta line) as ε_min_ decreases. Simultaneously, the number of determinants *N*_d_ increases with decreasing ε_min_. The system under consideration is O_2_ in the cc-pVDZ basis set. Other selection parameters are chosen as specified in Table 1.

The remaining tunable parameter ε_min_ controls the accuracy of the trial wave function. For all other parameters, we use the values listed in Table 1. Figure 4 illustrates the systematic convergence of the AFQMC/NOCI result to the reference value as ε_min_ decreases. At ε_min_ = 10^–6^, the trial wave function contains 119 determinants, reduces the error by 7.1 m*E*_h_, and agrees with the reference value within chemical accuracy.

**Table 1:** List of Optimal NOCI Selection Parameter Values Used throughout This Work, Unless Stated Otherwise^a^

*N*_*w*_	*N*_*k*_	τ	λ	μ	ε_max_	*N*_ξ_
6400	100	0.05 *E*_h_^–1^	4.0	0.6	≥10^–4^	10

a The ε_min_ parameter is the only remaining adjustable parameter that determines the accuracy of the NOCI selection.

**Figure 5:**
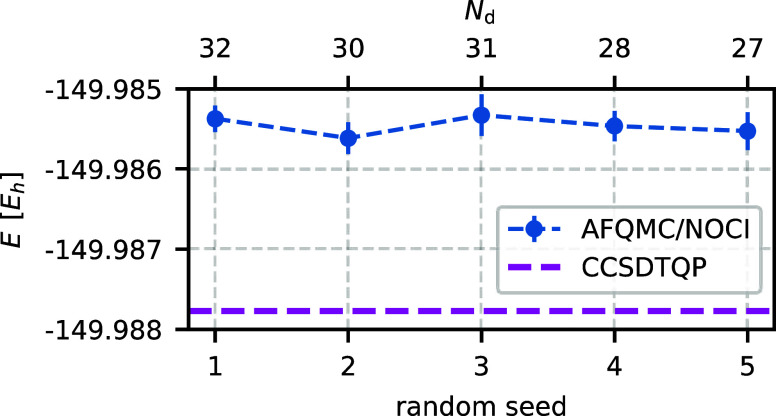
AFQMC/NOCI energy does not depend on the randomness of the NOCI selection process. Similarly, the number of determinants *N*_d_ remains nearly constant across all five random seeds used to initialize the AFQMC random walk. The system under consideration is O_2_ in the cc-pVDZ basis set. Other selection parameters are chosen as specified in Table 1, with ε_min_ = 10^–5^.

Finally, we demonstrate that the randomness of selecting the trial wave function does not impact its accuracy. We perform five identical selections with ε_min_ = 10^–5^, each using a different random seed. Figure 5 shows the AFQMC energies and their corresponding standard deviations for each random seed. The AFQMC energies are consistent within statistical errors, and the standard deviations remain nearly identical across all random seeds. Although the final number of determinants in the trial wave function varies slightly, as indicated on the top *x*-axis of Figure 5, this variation does not affect the accuracy or efficiency of the AFQMC simulation.

To demonstrate the transferability of the selection parameters listed in Table 1, we perform a similar analysis to that presented in Figures 1–5 for three additional systems: CN, N_2_ with equilibrium bond length *R*_0_, and N_2_ stretched to 2*R*_0_, as illustrated in Figure S1 of the Supporting Information. These results demonstrate that the choice of parameters is transferable and that the single parameter ε_min_ primarily governs the accuracy of the trial state as well as the number of determinants.

## Results and Discussion

In this section, we report AFQMC results using NOCI trial wave functions for the following cases: (i) second-row atoms, where the Hartree–Fock (HF) trial wave function performs poorly, (ii) HEAT set molecules,^23,59,60^ (iii) the benzene molecule^61^—a system with a dominant dynamic correlation, and (iv) the N_2_ dissociation as an example of strong static correlation effects.

We conduct all HF, NOCI, and AFQMC calculations using the QMCFort code^23^ with the cc-pVDZ basis set and the frozen-core approximation. For open-shell systems, we use the UHF (unrestricted HF) ground state in both NOCI selection and AFQMC calculations unless explicitly stated otherwise. The numerical parameters of the NOCI selection were described in detail in the previous section. We use modified Cholesky decomposition^62−64^ with a threshold of 10^–6^ to represent ERIs. Our large time-step algorithm^54^ enables a time step of 0.02 *E*_h_^–1^ without residual time-step errors. All AFQMC calculations employ 6400 walkers and run until the statistical error is below 0.2 m*E*_h_. Consequently, the figures showing AFQMC energies have no visible error bars. On the other hand, free-projection AFQMC calculations are performed using 64,000 walkers and 5000 time steps, with a time step of τ = 0.005 *E*_h_^–1^. All AFQMC values are provided in Tables S1–S4 of the Supporting Information.

### Second Row Atoms

While CCSD(T) energies for second-row isolated atoms are nearly exact, AFQMC with a HF trial wave function (AFQMC/HF) exhibits unexpectedly large errors.^23,36^ Therefore, single-determinant AFQMC could only predict quantities that do not involve the atomic energies of these elements. Consequently, these systems provide an ideal starting point for assessing the quality of the AFQMC using NOCI trial wave functions. We exclude the Li atom for which HF theory is already exact in the frozen-core approximation.

**Figure 6:**
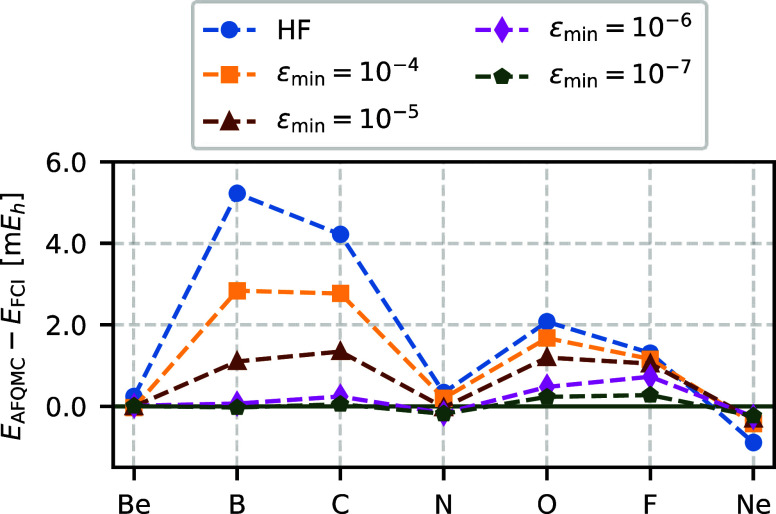
For second-row atoms, AFQMC/NOCI energies converge from AFQMC/HF toward the exact FCI energies as one tightens ε_min_. Calculations are performed using the cc-pVDZ basis set and frozen-core approximation.

Figure 6 depicts atomic AFQMC energies relative to FCI energies for the HF trial determinant and NOCI trial wave functions at several ε_min_ values. The AFQMC energies systematically converge toward the FCI values as ε_min_ decreases. The root-mean-square deviation (RMSD) reduces from 2.7 m*E*_h_ for the HF trial wave function to within chemical accuracy for the NOCI trial wave function at ε_min_ = 10^–5^. At this threshold, the NOCI selection yields an average of 25 Slater determinants. Tighter thresholds of ε_min_ = 10^–6^ or ε_min_ = 10^–7^ reduce the error to 0.4 and 0.2 m*E*_h_ with an average of 82 and 129 Slater determinants, respectively. More detailed statistics are provided in Table 2.

**Table 2:** Statistics for Second-Row Elements for AFQMC/HF and AFQMC/NOCI at Various ε_min_ Values with the cc-pVDZ Basis Set and Frozen-Core Approximation^a^

ε_min_	RMSD	MAD	max(|Δ*E*|)	⟨*N*_d_⟩	max(*N*_d_)
HF	2.7	2.0	5.2	1	1
10^–4^	1.7	1.3	2.8	5	9
10^–5^	0.9	0.7	1.3	25	34
10^–6^	0.4	0.3	0.7	82	111
10^–7^	0.2	0.1	0.3	129	179

a Root-mean-square deviation (RMSD), mean absolute deviation (MAD), and maximal absolute deviation max(|Δ*E*|) are presented in *E*_h_ units. ⟨*N*_d_⟩ is the average number of determinants for each ε_min_ value and max(*N*_d_) is the maximum number of determinants across all atoms for a given ε_min_ value.

### HEAT Set

The HEAT data set^59,60^ serves as a reliable benchmark for testing state-of-the-art correlation-consistent methods. We compare nearly exact CCSDTQP energies to AFQMC/HF, AFQMC/NOCI, and CCSD(T) ones. Although the largest system in the HEAT set (CO_2_ molecule) consists of 16 electrons in 39 orbitals, both CCSD(T) and AFQMC/HF have a relatively high RMSD of 1.7 and 2.9 m*E*_h_, respectively. This could be attributed to the fact that 14 systems are open-shell molecules, which are generally more challenging for quantum chemistry methods.

**Figure 7:**
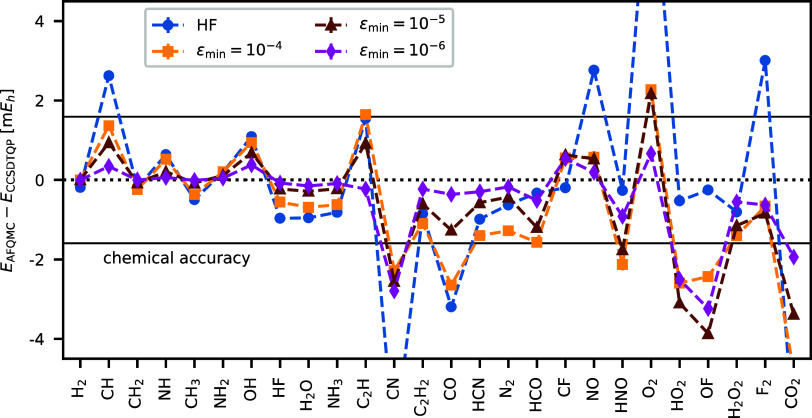
For HEAT set molecules, AFQMC/NOCI energies converge toward the CCSDTQP values as we tighten ε_min_. The RMSD decreases from 2.8 m*E*_h_ for AFQMC/HF to 1.1 m*E*_h_ for AFQMC/NOCI at ε_min_ = 10^–6^. Calculations are performed using the cc-pVDZ basis set and frozen-core approximation.

We calculate AFQMC/HF and AFQMC/NOCI energies at three different ε_min_ values. Figure 7 illustrates the errors in AFQMC/HF and AFQMC/NOCI energies at different ε_min_ values relative to the CCSDTQP values. The RMSD decreases from 2.8 m*E*_h_ for AFQMC/HF to 1.5 m*E*_h_ at ε_min_ = 10^–5^, and further to 1.1 m*E*_h_ at ε_min_ = 10^–6^. The average number of determinants in these calculations is 35 and 194, respectively. It is noteworthy that the residual errors are mainly associated with open-shell molecules. To demonstrate this, we calculate the RMSD for a subset of closed-shell HEAT molecules. For AFQMC/NOCI at ε_min_ = 10^–6^, the RMSD is reduced to 0.7 m*E*_h_ compared to 2.3 m*E*_h_ for AFQMC/HF. More detailed statistics are given in Table 3.

**Table 3:** Statistics for HEAT Set Molecules for AFQMC/HF and AFQMC/NOCI at Various ε_min_ Values, with the cc-pVDZ Basis Set and Frozen-Core Approximation^a^

ε_min_	RMSD	MAD	max(|Δ*E*|)	⟨*N*_d_⟩	max(*N*_d_)
HF	2.8	1.7	8.5	1	1
10^–4^	1.7	1.3	4.8	6	30
10^–5^	1.5	1.1	3.9	35	59
10^–6^	1.1	0.7	3.2	194	324

a Root-mean-square deviation (RMSD), mean absolute deviation (MAD), and maximal absolute deviation max(|Δ*E*|) are presented in *E*_h_ units. ⟨*N*_d_⟩ is the average number of determinants for each ε_min_ value and max(*N*_d_) represents the maximum number of determinants across all HEAT molecules for a given ε_min_ value.

We take a closer look at the molecules for which AFQMC/NOCI exhibits unexpected behavior. Specifically, we examine six molecules: CN, HCO, CF, HNO, HO_2_, and OF. In these cases, AFQMC/NOCI either underperforms compared to AFQMC/HF, or its performance deteriorates as the energy threshold ε_min_ decreases. Notably, five of these molecules are open-shell radicals, while only HNO is a closed-shell molecule. While HNO, like the isoelectronic O_2_, exhibits an open-shell ground state at the Hartree–Fock level, for post-Hartree–Fock methods, the closed-shell Hartree–Fock determinant is generally the preferred starting point.

We begin by investigating the difference between AFQMC energies using restricted HF (RHF) and unrestricted HF (UHF) trial wave functions. The AFQMC/RHF and AFQMC/UHF differ significantly only for the CN molecule, the only system in the HEAT set exhibiting strong spin contamination. For all other systems, RHF and UHF wave functions are of similar quality, with the UHF energies being only a few m*E*_h_ lower than the RHF values. Consequently, AFQMC/RHF and AFQMC/UHF yield a similar mean absolute deviation (MAD) of 1.3 and 1.4 m*E*_h_, respectively.

**Figure 8:**
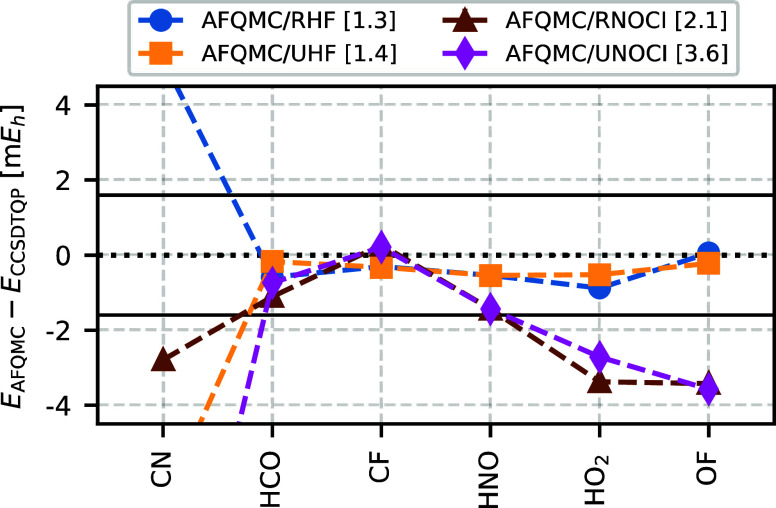
Restricted and unrestricted trial wave functions, both HF and NOCI, yield similar AFQMC energies for five out of six selected outliers from the HEAT set. The exception is the CN molecule, where the UHF wave function exhibits strong spin contamination and significantly deviates from the RHF wave function. As a result, AFQMC energies differ significantly between RHF and UHF, as well as between RNOCI and UNOCI trial wave functions. The numbers in square brackets represent the mean absolute deviation (MAD) of each method across the six selected HEAT molecules.

Next, we examine NOCI trial wave functions generated from RHF and UHF reference, denoted as RNOCI and UNOCI, respectively. In Figure 8, we see that AFQMC/RNOCI to AFQMC/UNOCI have larger deviations than the single Slater-determinant alternative. This leads to a larger MAD of 2.1 m*E*_h_ for AFQMC/RNOCI and 3.6 m*E*_h_ for AFQMC/UNOCI. The difference between AFQMC/RNOCI and AFQMC/UNOCI originates mostly from the CN molecule, for which AFQMC/RNOCI is much closer to the reference energy. For this reason, the RNOCI trial wave function was used for CN in Figure 7.

**Figure 9:**
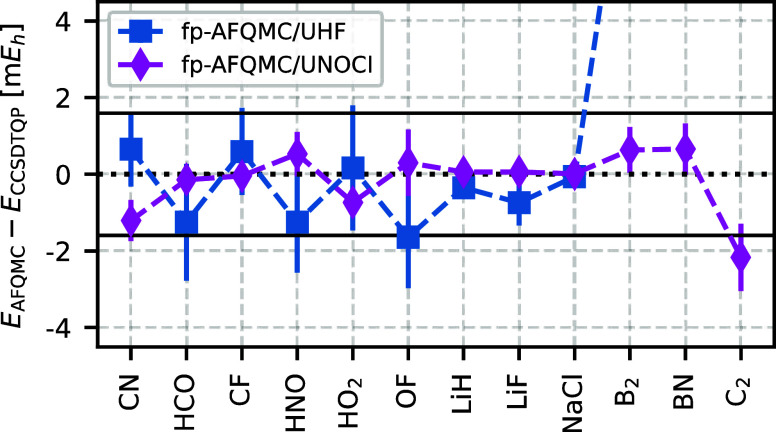
Free-projection (fp)-AFQMC using UHF and UNOCI trial wave functions yields more accurate energies than the corresponding ph-AFQMC calculations. However, the large statistical errors observed for UHF trial wave functions due to the phase problem highlight the advantage of UNOCI trial wave functions. Moreover, for strongly correlated systems such as B_2_, BN, and C_2_, we were unable to obtain accurate fp-AFQMC/UHF energies, while fp-AFQMC/UNOCI yields both small systematic and statistical errors.

To analyze the origin of these errors, we compare these results to free-projection AFQMC (fp-AFQMC) with UHF and UNOCI trial wave functions (see Figure 9). For these fp-AFQMC calculations, we employed the spin-projected AFQMC technique,^65^ which combines UHF or UNOCI trial wave functions with an RHF walker population. While this method does not affect the final AFQMC energy as long as the system is properly equilibrated, it significantly reduces the AFQMC equilibration time. As a result, it mitigates the onset of the phase problem, which is crucial for accumulating enough samples to estimate fp-AFQMC energies.

For the six outliers of the HEAT data set, fp-AFQMC/UHF yields an MAD of 0.9(6) m*E*_h_, slightly lower than that of AFQMC/UHF. However, the phase problem of the fp-AFQMC method leads to large error bars. In contrast, fp-AFQMC/UNOCI achieves a lower MAD of 0.5(2) m*E*_h_, more than seven times smaller than the corresponding AFQMC/UNOCI value. The better trial state seems to reduce the phase problem, leading to much smaller statistical errors. To further support this claim, we include six additional systems in Figure 9: three G1 molecules with strong ionic character (LiH, LiF, NaCl), for which Morales et al.^36^ observed slow convergence with respect to the number of nonorthogonal Slater determinants, and three strongly correlated W4-MR molecules (B_2_, BN, C_2_), for which Mahajan et al.^26^ reported the largest errors using AFQMC/CISD. For the first three molecules, both fp-AFQMC/UHF and fp-AFQMC/UNOCI yield almost exact energies with well-controlled statistical errors. In contrast, for the strongly correlated W4-MR molecules, only fp-AFMQC/UNOCI gives accurate results (within chemical accuracy), while fp-AFQMC/UHF even fails to properly equilibrate due to the onset of a severe phase problem. This again illustrates the advantage of the NOCI trial wave functions.

To summarize, these results demonstrate that the phaseless approximation is responsible for the large AFQMC/UNOCI errors. Notably, a higher quality trial wave function does not necessarily lead to smaller phaseless errors. Due to the ad hoc nature of the phaseless approximation, quantifying the dependence of the phaseless errors on the underlying trial wave function remains challenging. Our findings suggest that open-shell systems and strong spin contamination tend to exhibit larger AFQMC/NOCI errors compared to AFQMC/HF. Motivated by these observations, we investigate the dissociation of the N_2_ molecule.

### N_2_ Dissociation

The dissociation of the N_2_ molecule is a well-known example of strong static correlation effects caused by the breaking of the triple bond. The system also undergoes a symmetry-breaking transition, with a molecular-like RHF ground state at equilibrium geometry and an atomic-like UHF ground state at stretched geometries. The latter exhibits strong spin contamination and poses a significant challenge for AFQMC/NOCI.

We compute AFQMC total energies at six different bond lengths *R*, ranging from the equilibrium bond length of 2.118 *a*_0_ to 4.2 *a*_0_. We report AFQMC results for three trial wave functions: (i) the UHF determinant, denoted as AFQMC/UHF; (ii) the UNOCI wave function selected through the AFQMC random walk, denoted as AFQMC/UNOCI; and (iii) a special NOCI wave function obtained by diagonalizing only the RHF and two UHF determinants, denoted as AFQMC/RHF-UHF. Two UHF determinants are constructed by swapping the spin-up and spin-down orbitals. This trial wave function is motivated by the observation that both molecular and atomic-like solutions contribute significantly to the ground state near bond dissociation.

**Figure 10:**
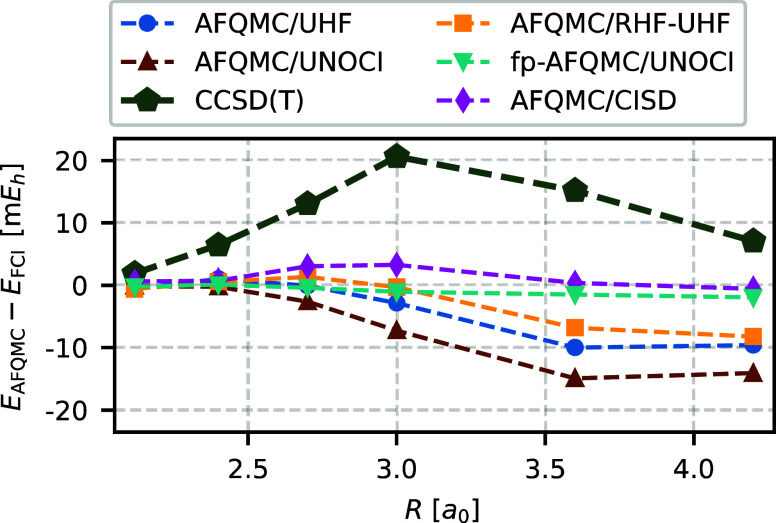
CCSD(T) energies and AFQMC energies with different trial wave functions, shown relative to the FCI energies. AFQMC/UNOCI performs worse than AFQMC/UHF for UHF trial wave functions with strong spin contamination. AFQMC/RHF-UHF yields nearly exact energies at intermediate bond lengths, where CCSD(T) and AFQMC/UNOCI exhibit the largest errors. Exact FCI energies are taken from ref (66), and AFQMC/CISD energies are taken from ref (26). All calculations are performed using the cc-pVDZ basis set and the frozen-core approximation.

Figure 10 shows the errors in our various AFQMC energies, CCSD(T) energies, and recently published AFQMC/CISD energies.^26^ The reference values are highly accurate DMRG energies taken from ref (66). CCSD(T) performs poorly with a maximal error of 20.6 m*E*_h_. In contrast, AFQMC/CISD achieves good accuracy, with a maximal error of approximately 4.0 m*E*_h_.

Our AFQMC/UHF energies agree well with those published in ref (26), yielding a maximal error of 10.0 m*E*_h_. Unfortunately, our AFQMC/UNOCI results are even less reliable than AFQMC/UHF, with an error of 14.6 m*E*_h_. We calculate fp-AFQMC/UNOCI energies to assess whether this mismatch is due to inappropriate UNOCI trial wave functions or phaseless errors. The maximal error of 2.0 m*E*_h_ demonstrates the quality of the UNOCI wave functions. This supports our hypothesis that AFQMC/UNOCI can exhibit phaseless errors larger than AFQMC/UHF for systems with strong spin contamination. In these simulations, we used RHF walkers to reduce the equilibration time, which allowed us to keep the statistical errors below 0.6 m*E*_h_ for all bond lengths. In contrast, we were unable to obtain meaningful fp-AFQMC results using UHF trial wave functions due to the severe phase problem.

In addition, AFQMC/RHF-UHF achieves nearly exact results at *R* = 2.7 *a*_0_ and *R* = 3.0 *a*_0_, which lie in the strongly correlated regime. Consequently, both CCSD(T) and AFQMC/CISD exhibit their largest errors at these bond lengths. This is an advantage of the AFQMC method, where compact trial wave functions, straightforwardly constructed using chemical intuition, outperform more sophisticated approaches that rely on the brute-force inclusion of determinants. However, AFQMC/RHF-UHF still fails at larger bond lengths, where the CI coefficient of the RHF determinant practically drops to zero. As a result of these large errors at stretched geometries, AFQMC/RHF-UHF produces errors up to 8.2 m*E*_h_.

### Benzene Molecule

The ground state of benzene, a singlet closed-shell configuration without near-degeneracies, is accurately represented by the Hartree–Fock determinant. However, the magnitude of the correlation energy is relatively large, amounting to −863 m*E*_h_ in the double-ζ basis set.^61^ Although the total correlation energy is dominated by dynamic correlation, both CCSD(T) and AFQMC/RHF exhibit notable errors. Specifically, CCSD(T) undercorrelates by 3.6 m*E*_h_, while AFQMC/RHF overcorrelates by 3.2 m*E*_h_.^23^ This makes benzene an excellent system to demonstrate the capabilities of post-Hartree–Fock quantum chemistry methods.

**Figure 11:**
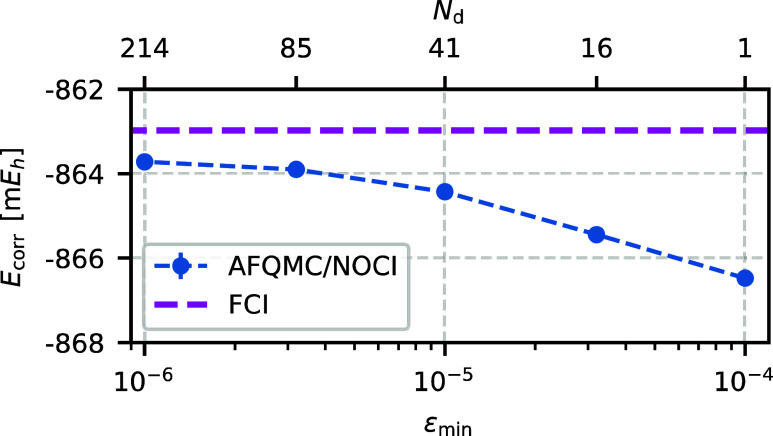
Correlation energy of benzene converges smoothly to the FCI value as ε_min_ decreases. Similarly, the number of determinants *N*_d_ increases with decreasing ε_min_, achieving chemical accuracy with only 41 Slater determinants. Calculations are performed using the cc-pVDZ basis set and frozen-core approximation.

We compute the benzene molecule with 30 electrons in 108 orbitals and compare AFQMC/NOCI to AFQMC/HF. Figure 11 illustrates that the AFQMC/NOCI correlation energy converges smoothly toward the FCI limit with tighter thresholds ε_min_. At ε_min_ = 10^–5^ (41 Slater determinants), the method reaches chemical accuracy with an error of 1.4 m*E*_h_. This error is reduced further to 0.7 m*E*_h_ at ε_min_ = 10^–6^, where the trial wave function contains 214 determinants.

Next, we compare our AFQMC energies with the previously published AFQMC values. Lee et al.^19^ reported AFQMC/RHF energies that agree with our AFQMC/RHF values within statistical error bars. They also performed AFQMC calculations using a CAS(6,6) trial wave function, where the full CAS expansion of 400 Slater determinants was truncated by discarding determinants with CI coefficients smaller than 10^–6^, resulting in 87 significant determinants.

**Figure 12:**
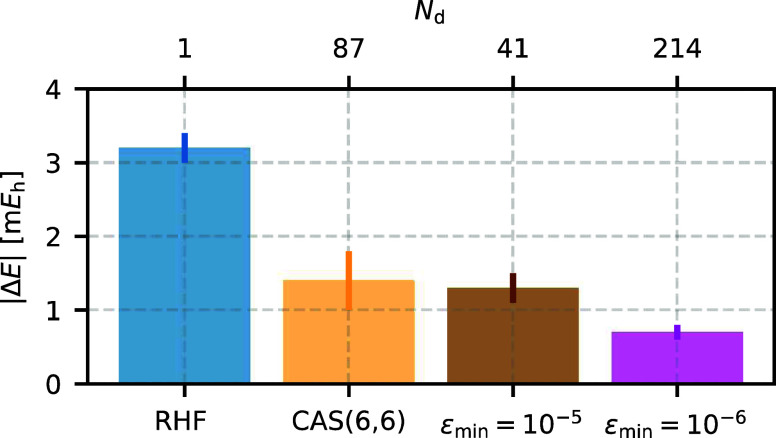
NOCI and CAS(6,6) trial wave functions effectively reduce the phaseless bias for the benzene molecule compared to the RHF trial wave function. Despite using fewer determinants, the AFQMC/NOCI trial wave function with ε_min_ = 10^–5^ (41 Slater determinants) achieves an accuracy similar to AFQMC/CAS(6,6), which includes 87 significant determinants in the CAS expansion. Lowering the energy threshold to ε_min_ = 10^–6^ results in the most accurate AFQMC energy for benzene reported to date.

Figure 12 summarizes these results and demonstrates that both CAS and NOCI trial wave functions effectively reduce the phaseless error. We find that the AFQMC/NOCI trial wave function with 41 determinants (ε_min_ = 10^–5^) achieves an accuracy comparable to the CAS(6,6) trial wave function with 87 determinants. Increasing the number of NOCI determinants to 214, by tightening the energy threshold to ε_min_ = 10^–6^, further reduces the phaseless error and yields the most accurate AFQMC energy for the benzene molecule reported to date.

## Performance and Scaling Analysis of AFQMC/NOCI

In this section, we systematically benchmark the accuracy and performance of the AFQMC/NOCI method. We begin by analyzing the size-consistency of AFQMC/NOCI energies, followed by the analysis of the time-step errors. Finally, we investigate the computational cost scaling of AFQMC/NOCI with respect to the number of determinants in the trial wave function and determine conditions under which AFQMC/NOCI becomes a more efficient alternative to conventional AFQMC/HF.

### Size-Consistency

An important property of any quantum chemistry method is size-consistency. A method is size-consistent if the total energy of two noninteracting fragments *A* and *B* equals the sum of their individual energies, i.e., *E*(*A* + *B*) = *E*(*A*) + *E*(*B*). This property ensures that the method correctly describes systems in the dissociation limit. This is essential for accurately predicting binding and reaction energies.

While exact full configuration interaction (FCI) is guaranteed to be size-consistent, several approximate methods, such as Hartree–Fock and coupled-cluster theory, also satisfy this requirement. Lee et al.^22^ proved that AFQMC/HF is size-consistent in the limit of vanishing time steps. We have recently shown that AFQMC/HF retains size-consistency for large time steps, provided that rare events are carefully controlled.^54^

In contrast, truncated CI methods are generally not size-consistent. This includes the NOCI approach we used throughout this work to refine AFQMC trial wave functions. Therefore, we investigate whether the size inconsistency of the NOCI method taints the size consistency of the AFQMC/NOCI. To this end, we examine noninteracting chains of N_2_ molecules in the cc-pVDZ basis set.

**Figure 13:**
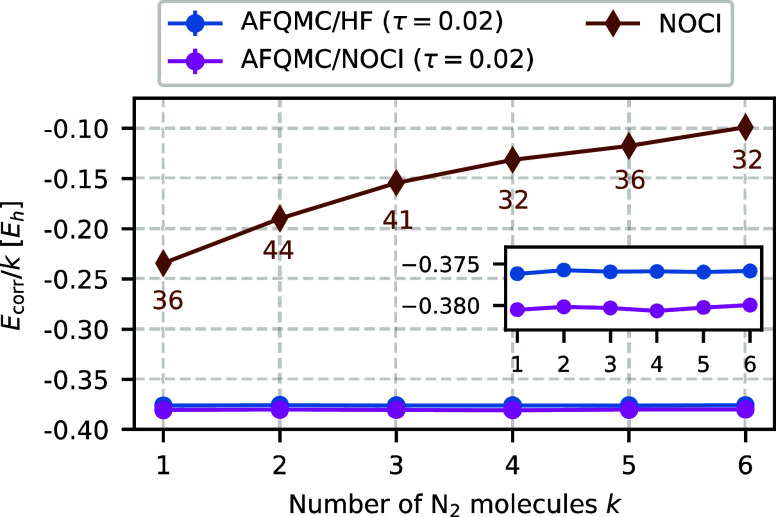
AFQMC/NOCI correlation energies (magenta line) per N_2_ molecule are independent of the number of molecules *k*, demonstrating size-consistency, even though the underlying NOCI trial wave functions (brown line) are not. The inset provides a zoomed-in comparison between the size-consistent AFQMC/HF (blue line) and AFQMC/NOCI energies. The numbers below the brown line indicate the number of determinants in the NOCI trial wave function for each *k*.

Figure 13 shows the correlation energy per N_2_ molecule as a function of the number of molecules *k* in the chain. As expected, AFQMC/HF (blue line) is size-consistent. Remarkably, AFQMC/NOCI (magenta line) also yields size-consistent correlation energies, even though the NOCI trial wave functions themselves are not size-consistent (brown line). It is also noteworthy that the number of determinants in the NOCI expansions remains nearly constant across all chain lengths *k*.

### Time-Step Errors

Next, we examine time-step errors of the AFQMC method by analyzing the total energies of the N_2_ molecule, and binding energies of the water dimer, both in the cc-pVDZ basis set.

**Figure 14:**
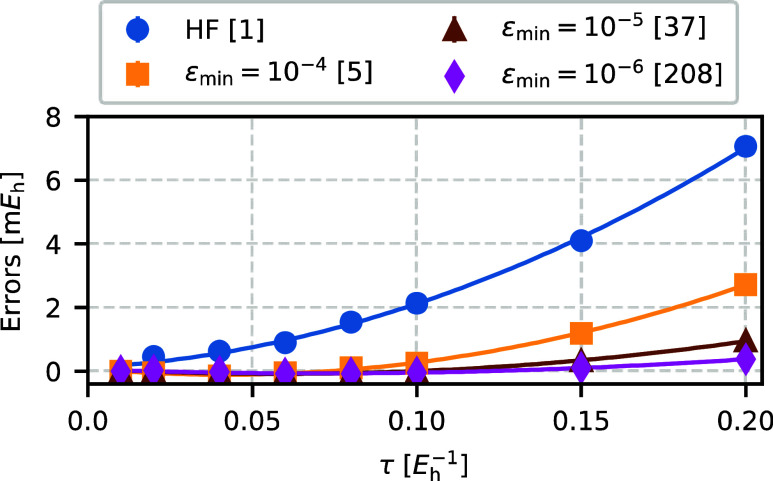
Time-step errors in the AFQMC total energy for N_2_ in the cc-pVDZ basis set. Errors decrease significantly as ε_min_ tightens. For ε_min_ = 10^–6^, time-step errors become negligible for all time steps up to τ = 0.20 *E*_h_^–1^. The numbers in square brackets indicate the number of Slater determinants in the corresponding trial wave functions.

Figure 14 shows time-step errors in the total AFQMC energy of the N_2_ molecule for the HF trial wave function and AFQMC/NOCI trials at several ε_min_ values. As ε_min_ decreases, time-step errors decrease as well. For ε_min_ = 10^–6^ (208 Slater determinants), time-step errors are essentially negligible for all considered time steps.

**Figure 15:**
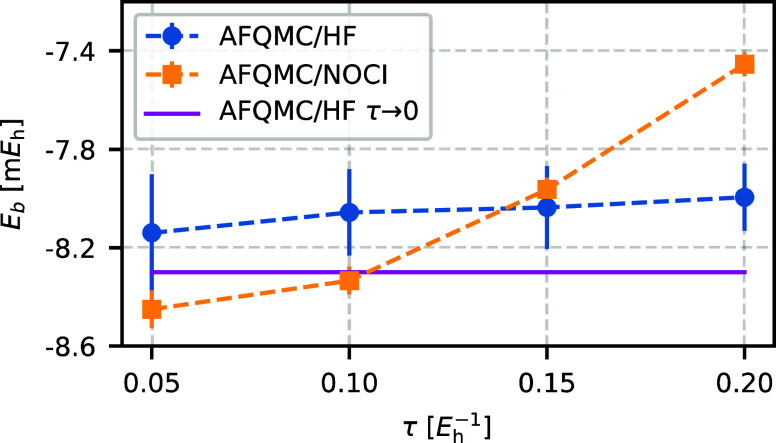
Time-step errors in AFQMC binding energies are larger for AFQMC/NOCI compared to AFQMC/HF due to the imperfect cancellation of the time-step errors between NOCI trial wave functions. For the water dimer in the cc-pVDZ basis set, AFQMC/NOCI exhibits larger time-step errors for τ ≥ 0.10 *E*_h_^–1^. The NOCI trial wave function was generated using ε_min_ = 10^–5^, yielding 37 Slater determinants for the water monomer and 36 for the dimer.

Next, Figure 15 shows the binding energies of the water dimer as a function of the time step for both AFQMC/HF and AFQMC/NOCI trial wave function at ε_min_ = 10^–5^ containing 37 Slater determinants for the water monomer and 36 determinants for the dimer. For small time steps, both methods yield consistent binding energies within statistical error bars. However, for larger time steps (τ > 0.10 *E*_h_^–1^), AFQMC/NOCI shows larger errors. We attribute this behavior to the NOCI selection process. Since the HF determinant is a unique choice, errors in different geometries can benefit from error cancellation. In contrast, the NOCI selection may yield different quality trial wave functions for different geometries which impedes this error cancellation.

In summary, improved trial wave functions reduce time-step errors in total energies, enabling the use of larger time steps in AFQMC simulations. However, for relative energies such as binding energies, AFQMC/NOCI may show larger time-step errors due to imperfect cancellation of time-step errors between different NOCI trial wave functions.

### Computational Scaling with Number of Determinants

For a fixed number of AFQMC steps, the computational cost of AFQMC/NOCI, denoted as *T*_NOCI_, scales linearly with the number of determinants *N*_d_ in the trial wave function, relative to the cost of AFQMC/HF *T*_HF_
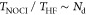
25However, it is more relevant to examine how the AFQMC/NOCI method scales with *N*_d_ for fixed variance. To account for this, we consider the quantity , where σ represents the standard deviation of the AFQMC local energies corresponding to a given trial wave function |Φ_*N*_d__⟩. For the purpose of this analysis, we use the empirically observed relationship σ ≈ |*E*_0_ – *E*_*N*_d__|, with supporting data in Figure S2 of the Supporting Information. Here, *E*_0_ is the exact ground-state energy of the system, and  is the trial energy.

Furthermore, we assume that the correlation energy in the NOCI state converges as
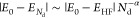
26where α is the NOCI convergence exponent. This finally leads to
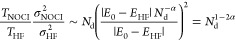
27This result suggests that the overall scaling of AFQMC/NOCI is determined by the NOCI convergence exponent α. Notably, it implies that for α > 0.5, AFQMC/NOCI becomes more efficient than AFQMC/HF.

**Figure 16:**
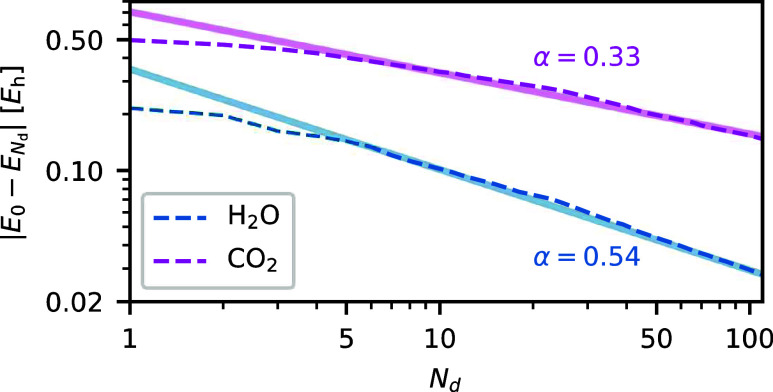
Missing correlation energy |*E*_0_ – *E*_*N*_d__| of the NOCI wave function |Φ_*N*_d__⟩ decreases sublinearly with the number of determinants *N*_d_, following a power law governed by an exponent α. This exponent determines the efficiency of the AFQMC/NOCI procedure. For α ≥ 0.5, AFQMC/NOCI becomes more efficient than the conventional AFQMC/HF at fixed variance.

To verify our hypothesis, we first determine the exponent α for two representative molecules from the HEAT set, as illustrated in Figure 16. Extending the analysis to 25 molecules from the HEAT set, we observe an average exponent of α = 0.46 ± 0.14—close to the critical value of 0.5. Similar α values are observed for N_2_ at different bond lengths, indicating that the analysis is also valid for strongly correlated systems.

**Figure 17:**
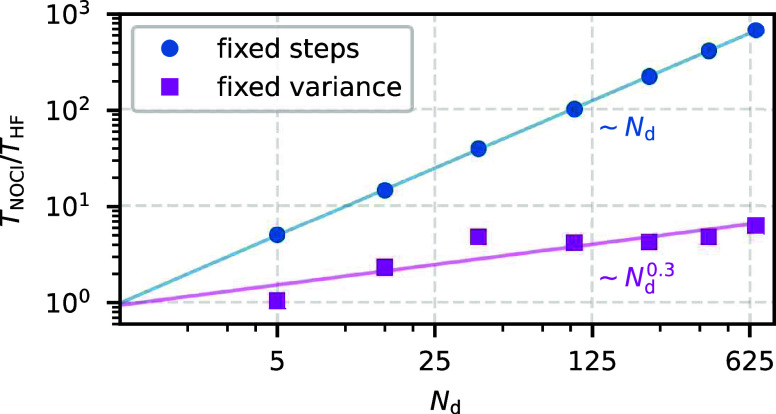
AFQMC/NOCI wall time for a fixed number of AFQMC steps scales linearly with the number of determinants. In contrast, for a fixed variance, the scaling becomes sublinear, following a power law with an exponent of 1 – 2α, where α characterizes the convergence of the NOCI correlation energy. For the specific case of the N_2_ molecule in the cc-pVDZ basis set, we observe *T*_NOCI_/*T*_HF_ ∼ *N*_d_^0.3^. As a result, an AFQMC/NOCI simulation with 664 determinants is only eight times more expensive than the corresponding AFQMC/HF calculation.

Finally, Figure 17 illustrates the scaling behavior of the AFQMC/NOCI method on the example of the N_2_ molecule, for which we estimated α = 0.34. The blue curve shows the ratio *T*_NOCI_/*T*_HF_ for a fixed number of AFQMC steps, confirming the expected linear scaling with respect to *N*_d_. In contrast, the magenta curve shows *T*_NOCI_/*T*_HF_ for a fixed statistical variance, revealing sublinear scaling ∼*N*_d_^0.3^, which is in excellent agreement with the predicted behavior ∼*N*_d_^1–2α^, where 1 – 2α = 0.32.

## Conclusions

In this work, we combined the nonorthogonal configuration interaction (NOCI) method with the phaseless auxiliary-field quantum Monte Carlo (AFQMC) random walk to generate more accurate trial wave functions for AFQMC. We introduced an efficient selection algorithm to identify the most important AFQMC random walkers based on three criteria: (i) low local energies, (ii) small overlap with the currently sampled trial wave function, and (iii) a significant lowering of the variational energy of the trial wave function. We used the O_2_ molecule, a system where AFQMC/HF performs poorly, to calibrate the algorithm. Most parameters of the selection algorithm have minimal impact on its accuracy and can be set to reasonable default values. In the end, a single adjustable parameter ε_min_ governs the accuracy and the number of determinants in the sampled trial wave function. We showed that AFQMC/NOCI systematically converges to the reference value as we tighten ε_min_.

We demonstrated that the NOCI trial wave function improves AFQMC to achieve chemical accuracy for atoms and the HEAT set. For second-row elements, AFQMC/NOCI reduces the RMSD compared to AFQMC/HF by a factor of 10 using an average of 129 Slater determinants. Similarly, an average of 35 determinants obtained with ε_min_ = 10^–5^ are sufficient to reduce the RMSD for the HEAT molecules to less than 1 kcal/mol. For the benzene molecule, we reduced the AFQMC errors by 80% with a NOCI trial wave function of 214 determinants.

In addition, we show that AFQMC/NOCI yields size-consistent energies, although the underlying trial wave functions are not strictly size-consistent. Furthermore, the NOCI wave functions significantly reduce AFQMC time-step errors. Finally, we found that the computational cost of AFQMC/NOCI scales sublinearly with the number of determinants, following a power law whose exponent α is fully determined by the convergence rate of the correlation energy recovered by the NOCI trial wave function. Notably, for exponents α > 0.5, AFQMC/NOCI becomes more efficient than conventional AFQMC/HF, because it significantly reduces the AFQMC sampling variance.

For strongly correlated systems, phaseless errors do not always decrease smoothly with improved NOCI trial wave functions. Using the N_2_ dissociation as an example, we show that AFQMC/NOCI can sometimes lead to larger errors than AFQMC/HF. The near-exact free-projection AFQMC/NOCI energies across all bond lengths suggest that the dominant source of error arises from the phaseless approximation itself. Therefore, future work should focus on improving the AFQMC/NOCI for strongly correlated systems. Two promising directions are (i) further optimization of the NOCI trial wave functions, potentially including orbital rotations, and (ii) a systematic revision of the phaseless approximation.

However, for weakly correlated systems, our selection algorithm, based on AFQMC random walks, provides a systematic approach to refine AFQMC trial wave functions without the need for another approach to determine multideterminantal trial wave functions. We have shown that trial states with 100–200 nonorthogonal Slater determinants achieve AFQMC energies within chemical accuracy for all weakly correlated systems analyzed in this work. This is particularly exciting for solid-state systems, where sophisticated many-body approaches are often not yet available or computationally still very expensive.

## Supporting Information Available

Raw AFQMC energies (Tables S1–S4); demonstrating the transferability of the NOCI selection parameters (Figure S1), and illustrating the relationship between the standard deviation of the local energy and the correlation energy (Figure S2) (PDF)
