# Supplementary material for: Self-Refinement of Auxiliary-Field Quantum Monte Carlo via Non-Orthogonal Configuration Interaction
Source: J Chem Theory Comput. 2025 Apr 28;21(9):4481–93. doi: 10.1021/acs.jctc.5c00127 (PMC12080107; doi:10.1021/acs.jctc.5c00127)
Supplement: Supplementary file 1 — ct5c00127_si_001.pdf [file ct5c00127_si_001.pdf]

Supplementary Material for: "Self-Refinement of Auxiliary-Field Quantum Monte Carlo via Non-Orthogonal Configuration Interaction"

Zoran Sukurma,[*,†] Martin Schlipf,[*,‡] and Georg Kresse[*,†,‡]

†*University of Vienna, Faculty of Physics, Kolingasse 14-16, A-1090 Vienna, Austria*

‡*VASP Software GmbH, Berggasse 21/14, 1090 Vienna, Austria*

E-mail: zoran.sukurma@univie.ac.at; martin.schlipf@vasp.at; georg.kresse@univie.ac.at

Abstract

This file contains the supplementary information for the paper titled "Self-Refinement of Auxiliary-Field Quantum Monte Carlo via Non-Orthogonal Configuration Interaction". The numerical values provided in Tables 1, 2, and 3 correspond to the data plotted in Figures 6, 7, and 9 in the main paper, respectively. The general calculation setup is described at the beginning of the results section in the main paper.

Transferability of NOCI Selection Parameters

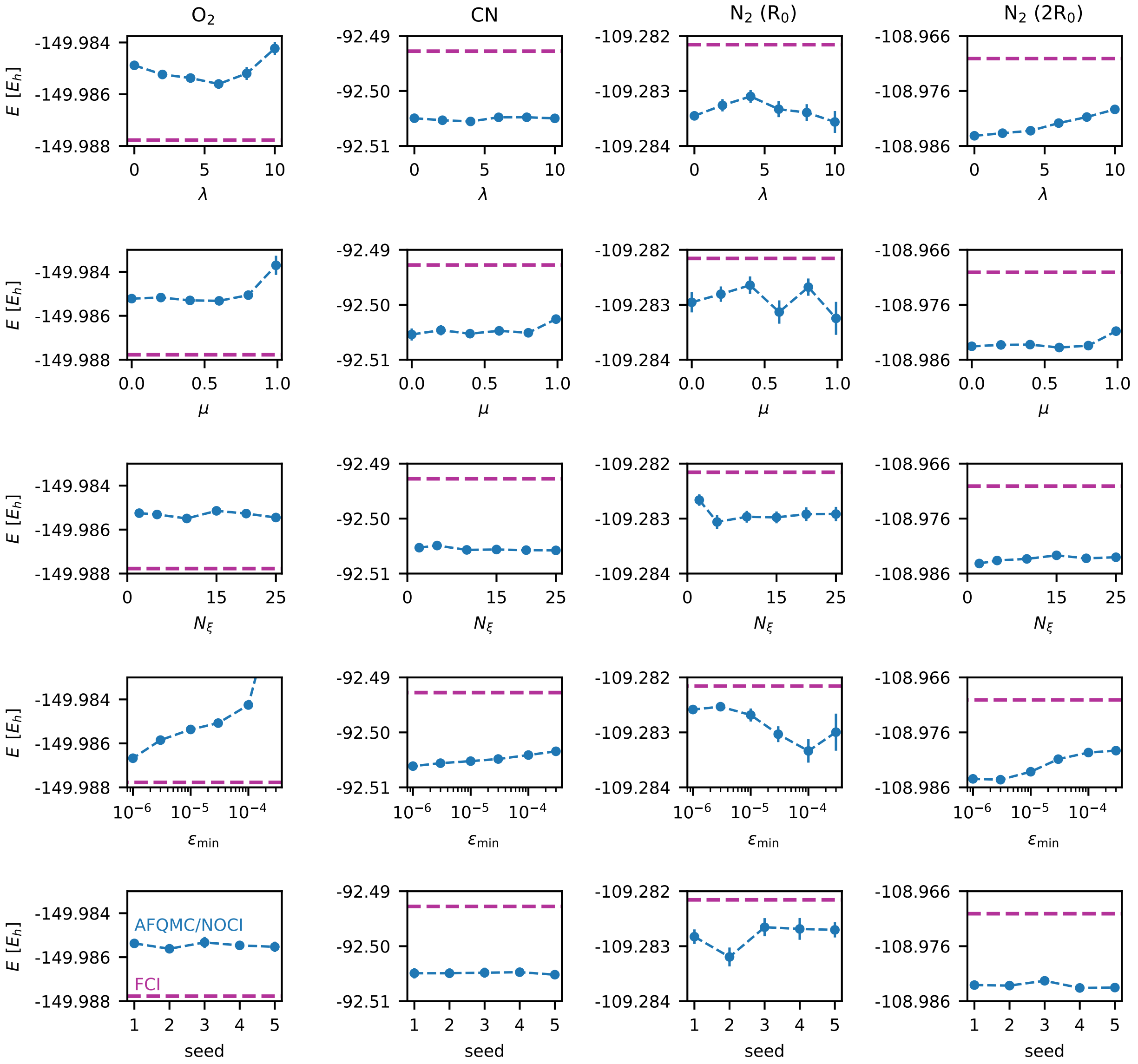

Figure S1: The NOCI selection parameter scan shows that the chosen parameters are transferable across different systems. It also demonstrates that the parameter ε_{min} (fourth row) primarily determines the accuracy of the selected trial wave function. We examined two examples of weakly correlated systems (O_2 and N_2 at equilibrium bond length R_0), one example with the moderate static correlation (CN), and one strongly correlated system (N_2 at stretched bond length $2R_0$).

Relationship between Local Energy Sampling Variance and the Correlation Energy

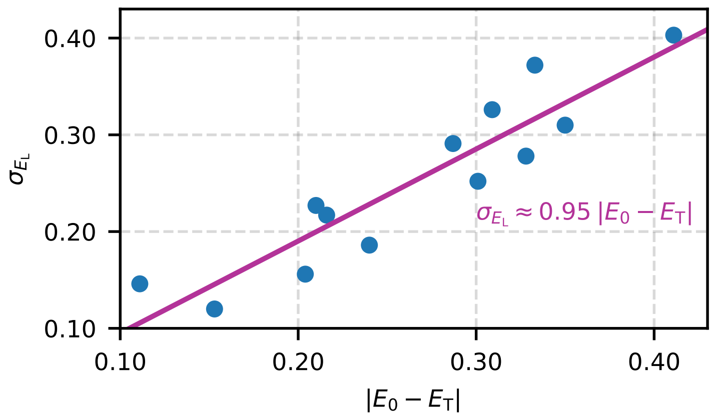

Figure S2: The local energy standard deviation σ_{E_L} is proportional to the missing correlation energy $|E_0 - E_T|$ of the trial wave function.

Second-Row Atoms

Table S1: AFQMC total energies (in Hartree units) for the second-row atoms using the Hartre-Fock trial wave function and NOCI trial wave functions at several ε_{min} values. Reference values are taken from Ref. 1. The last two rows of the table depict the root-mean-square deviation (RMSD), and mean absolute deviation (MAD).

Atom	AFQMC/HF	ε_{min}=10^{-4}	ε_{min}=10^{-5}	ε_{min}=10^{-6}	ε_{min}=10^{-7}
Be	−14.616 60(9)	−14.616 87(3)	−14.616 850(8)	−14.616 829(4)	−14.616 387(3)
B	−24.584 179(7)	−24.586 57(4)	−24.588 31(2)	−24.589 342(8)	−24.589 441(7)
C	−37.756 16(7)	−37.757 61(4)	−37.759 03(2)	−37.760 140(9)	−37.760 327(6)
N	−54.478 16(6)	−54.478 31(3)	−54.478 54(1)	−54.478 674(4)	−54.478 685(3)
O	−74.907 87(6)	−74.908 27(4)	−74.908 75(2)	−74.909 473(8)	−74.909 721(8)
F	−99.526 27(8)	−99.526 42(4)	−99.526 53(2)	−99.526 85(1)	−99.527 299(8)
Ne	−128.679 73(9)	−128.679 26(4)	−128.679 15(1)	−128.679 095(6)	−128.679 074(5)
RMSD	0.0027	0.0017	0.0009	0.0004	0.0002
MAD	0.0020	0.0013	0.0007	0.0003	0.0001

HEAT Set

Table S2: AFQMC total energies (in Hartree units) for 26 molecules in the HEAT[2] set, calculated using the Hartree-Fock trial wave function and NOCI trial wave functions at different $\varepsilon_{\rm min}$ values. Reference CCSDTQP values are obtained from Ref. 3. The last two rows of the table depict the root-mean-square deviation (RMSD), and mean absolute deviation (MAD).

Molecule	AFQMC/HF	$\varepsilon_{\rm min}$=10^{-4}	$\varepsilon_{\rm min}$=10^{-5}	$\varepsilon_{\rm min}$=10^{-6}
H_2	−1.163613(12)	−1.163429(4)	−1.163423(4)	−1.163429(2)
CH	−38.377617(32)	−38.378874(59)	−38.379304(39)	−38.379898(13)
CH_2	−39.041843(24)	−39.041892(42)	−39.041728(15)	−39.041652(8)
NH	−55.091062(27)	−55.091178(62)	−55.091493(19)	−55.091638(7)
CH_3	−39.716564(30)	−39.716440(56)	−39.716150(21)	−39.716080(8)
NH_2	−55.732888(36)	−55.732871(48)	−55.732963(24)	−55.733030(8)
OH	−75.558597(34)	−75.558744(56)	−75.559008(22)	−75.559307(10)
HF	−100.229586(38)	−100.229179(52)	−100.228850(21)	−100.228698(7)
H_2O	−76.242602(45)	−76.242338(57)	−76.241932(23)	−76.241802(13)
NH_3	−56.403332(42)	−56.403140(60)	−56.402739(30)	−56.402606(9)
C_2H	−76.399673(59)	−76.39957(11)	−76.40030(15)	−76.40145(10)
CN	−92.499413(72)	−92.49504(11)	−92.495312(80)	−92.495552(71)
C_2H_2	−77.111531(85)	−77.11178(14)	−77.111283(80)	−77.110907(28)
CO	−113.059086(79)	−113.05854(11)	−113.057157(58)	−113.056257(31)
HCN	−93.190896(89)	−93.191310(11)	−93.190483(67)	−93.190208(34)
N_2	−109.277639(87)	−109.27829(11)	−109.277444(53)	−109.277186(22)
HCO	−113.577712(73)	−113.57895(11)	−113.578582(73)	−113.577902(41)
CF	−137.476217(59)	−137.475465(92)	−137.475390(95)	−137.475480(48)
NO	−129.596971(77)	−129.59916(16)	−129.599208(66)	−129.599547(35)
HNO	−130.173171(86)	−130.17503(15)	−130.17466(10)	−130.173821(46)
O_2	−149.979264(74)	−149.98550(10)	−149.985600(62)	−149.987109(22)
HO_2	−150.560906(68)	−150.56297(13)	−150.563479(94)	−150.562888(53)
OF	−174.500342(71)	−174.50252(13)	−174.503957(76)	−174.503328(53)
H_2O_2	−151.196070(76)	−151.19667(13)	−151.196418(65)	−151.195819(45)
F_2	−199.096322(90)	−199.09999(20)	−199.100165(73)	−199.099958(37)
CO_2	−188.155912(97)	−188.15437(13)	−188.152933(80)	−188.151493(61)
RMSD	0.0028	0.0017	0.0015	0.0011
MAD	0.0017	0.0013	0.0011	0.0007

N_2 Dissociation

Table S3: Various AFQMC energies for the N_2 molecule at different bond lengths relative to the nearly exact DMRG energies.[4]

R $[a_0]$	AFQMC/UHF	AFQMC/RHF-UHF	AFQMC/UNOCI	fp-AFQMC/UNOCI
2.118	−0.59(16)	−0.59(16)	−0.12(5)	−0.26(13)
2.4	1.01(13)	0.56(12)	−0.23(5)	0.08(16)
2.7	−0.07(11)	1.33(12)	−2.62(6)	−0.40(26)
3.0	−2.83(11)	−0.30(11)	−7.26(9)	−1.06(35)
3.6	−9.96(7)	−6.80(8)	−14.91(15)	−1.51(63)
4.2	−9.57(6)	−8.22(6)	−14.08(14)	−1.97(23)

Benzene Molecule

Table S4: AFQMC correlation energies (in Hartree units) for the benzene molecule using the Hartre-Fock trial wave function and NOCI trial wave functions at different ε_{min} values. The Hartree-Fock energy $E_{\text{HF}} = -230.721818$ E_h is calculate using the QMCFort[5] code, while the exact correlation energy $E_{\text{corr}} = -863.0$ mE_h is obtained from Ref. 6.

ε_{min}	E_{corr} [mE_h]
HF	−866.47(18)
3.2×10^{-5}	−865.44(18)
10^{-5}	−864.43(18)
3.2×10^{-6}	−863.90(15)
10^{-6}	−863.72(11)

References

(1) Mahajan, A.; Thorpe, J. H.; Kurian, J. S.; Reichman, D. R.; Matthews, D. A.; Sharma, S. Beyond CCSD(T) Accuracy at Lower Scaling with Auxiliary Field Quantum Monte Carlo. *J.Chem. Theory Comput.* **2025**, *21*, 1626–1642, PMID: 39907123.

(2) Tajti, A.; Szalay, P. G.; Császár, A. G.; Kállay, M.; Gauss, J.; Valeev, E. F.; Flowers, B. A.;

Vázquez, J.; Stanton, J. F. HEAT: High accuracy extrapolated ab initio thermochemistry. *J. Chem. Phys.* **2004**, *121*, 11599–11613.

(3) Bomble, Y. J.; Stanton, J. F.; Kállay, M.; Gauss, J. Coupled-cluster methods including noniterative corrections for quadruple excitations. *J. Chem. Phys.* **2005**, *123*, 054101.

(4) Chan, G. K.-L.; Kállay, M.; Gauss, J. State-of-the-art density matrix renormalization group and coupled cluster theory studies of the nitrogen binding curve. *J. Chem. Phys.* **2004**, *121*, 6110–6116.

(5) Sukurma, Z.; Schlipf, M.; Humer, M.; Taheridehkordi, A.; Kresse, G. Benchmark Phaseless Auxiliary-Field Quantum Monte Carlo Method for Small Molecules. *J. Chem. Theory Comput.* **2023**, *19*, 4921–4934.

(6) Eriksen, J. J.; Anderson, T. A.; Deustua, J. E.; Ghanem, K.; Hait, D.; Hoffmann, M. R.; Lee, S.; Levine, D. S.; Magoulas, I.; Shen, J.; et al. The Ground State Electronic Energy of Benzene. *J. Phys. Chem. Lett.* **2020**, *11*, 8922–8929, PMID: 33022176.
